# Physiological Monitoring of Sound-Based Relaxation Using Binaural Audio and Vibroacoustic Stimulation

**DOI:** 10.3390/s26144391

**Published:** 2026-07-10

**Authors:** Joel Preto Paulo, António Fernandes, André Lourenço

**Affiliations:** 1 Instituto Superior de Engenharia de Lisboa (ISEL), Instituto Politécnico de Lisboa, R. Conselheiro Emídio Navarro 1, 1959-007 Lisboa, Portugal; antonio.nfernandes@isel.pt; 2 LAA—Audio and Acoustics Laboratory, Instituto Superior de Engenharia de Lisboa (ISEL), 1959-007 Lisboa, Portugal; 3 NOVA Laboratory for Computer Science and Informatics (NOVA LINCS), Instituto Superior de Engenharia de Lisboa (ISEL), 1959-007 Lisboa, Portugal; 4 CardioID Technologies, Parque Tecnológico de Óbidos, R. da Criatividade, 2510-230 Óbidos, Portugal

**Keywords:** sound interventions, binaural beats, immersive sound, physiological signals, relaxation assessment, sensor fusion

## Abstract

Immersive audio and vibroacoustic stimulation have gained increasing attention as non-invasive approaches for modulating human emotional and physiological states. The SonikB3D platform was previously introduced as a multisensory system combining immersive 3D audio, vibroacoustic stimulation, and physiological monitoring. Building upon this prior work, the present study advances the platform through a refined experimental protocol and a data-driven framework for the automatic assessment of relaxation using multimodal biosignals. A controlled pilot study was conducted with 20 participants exposed to 3D sound and vibroacoustic stimulation delivered through a massage table equipped with integrated transducers. Although the SonikB3D platform supports multiple stimulation scenarios, the present study focuses on a single controlled condition combining binaural 3D audio (binaural beats plus music) and vibroacoustic stimulation in order to ensure methodological consistency for multimodal modelling. Physiological responses were continuously recorded using a synchronized setup including electroencephalography (EEG), photoplethysmography (PPG), and electrodermal activity (EDA). Subjective emotional self-assessment questionnaires were collected before and after exposure to provide a multidimensional characterization of participant responses. Results show a statistically significant increase in self-reported relaxation (paired *t*-test = 3.05, *p* = 0.01), corresponding to an average 8% improvement in normalized relaxation scores. To support objective assessment, multimodal physiological features associated with autonomic and emotional regulation were extracted and used to develop a two-stage machine learning pipeline. The proposed model, combining a window-level Random Forest classifier with session-level aggregation, achieved an accuracy of 80% and an F1-score of 0.857 in classifying relaxation-related states. These findings provide preliminary evidence that combined 3D audio and vibroacoustic stimulation can produce measurable changes in subjective and physiological indicators of relaxation, while demonstrating the feasibility of automatic relaxation state inference from multimodal biosignals. Although exploratory due to the limited sample size and the absence of unimodal control conditions, this work contributes a data-driven methodology for studying human responses to multisensory sound and vibration metrics.

## 1. Introduction

Sound-based interventions have long been used across cultures as tools for relaxation, meditation, and emotional regulation [1,2,3]. Advances in neuroscience and psychoacoustics have enabled a more rigorous understanding of how auditory stimulation influences both the central and autonomic nervous systems [4]. Experimental evidence indicates that sound stimuli can modulate physiological responses related to stress, emotional regulation n, and well-being [5,6].

Vibroacoustic stimulation extends auditory perception by introducing a tactile component, allowing sound to be perceived simultaneously through hearing and body vibrations. This multisensory approach has attracted increasing attention in therapeutic and wellness contexts, particularly for relaxation and stress reduction [7,8,9]. At the same time, the availability of wearable physiological sensors has enabled continuous monitoring of cardiovascular, electrodermal, and neural activity, providing objective indicators of emotional and physiological states [10].

Despite these advances, existing research typically investigates auditory stimulation or vibroacoustic stimulation in isolation, and relatively few studies combine immersive 3D audio, synchronized vibroacoustic stimulation, and multimodal physiological monitoring within a unified experimental framework [11,12].

EEG has been extensively used to investigate oscillatory dynamics associated with relaxation, meditation and emotional regulation [13,14,15]. Autonomic markers such as HRV and EDA are well-established indices of autonomic balance and stress [16]. Despite this toolbox, few studies integrate machine learning and autonomic recordings with precisely engineered multisensory stimulation in immersive contexts.

To address these gaps, this paper uses SonikB3D, a multisensory platform designed to investigate the physiological and subjective effects of combined auditory and vibroacoustic stimulation. The system integrates vibroacoustic transducers embedded in a modular therapeutic bed with immersive 3D audio reproduction, delivered either through headphones or a loudspeaker array in an immersive sound room. The platform is coupled with synchronized physiological monitoring that records electroencephalography (EEG), photoplethysmography (PPG), and electrodermal activity (EDA), enabling comprehensive assessment of autonomic and neurophysiological responses during sound stimulation sessions [17].

Using this platform, we analyze both objective and subjective indicators of relaxation. Objective measures include heart-rate variability metrics derived from PPG, electrodermal activity markers of sympathetic arousal, and EEG spectral features associated with cortical relaxation. Subjective perceptions of relaxation are assessed using self-report questionnaires administered before and after each session, providing complementary insight into participants’ perceived emotional states.

A preliminary version of the SonikB3D platform was previously introduced in a conference study, where the feasibility of combining immersive 3D audio, vibroacoustic stimulation and physiological monitoring was demonstrated through an exploratory experimental protocol and threshold-based physiological indicators. The present work substantially extends this earlier study by introducing an expanded dataset, a refined experimental protocol, advanced multimodal feature extraction, and a machine learning framework for the automatic assessment of relaxation-related states.

The main contributions of this work are:

- Experimental validation of a multisensory immersive sound and vibration platform using synchronized EEG, PPG and EDA monitoring in human participants;
- Collection and analysis of a multimodal physiological dataset acquired from human participants during controlled immersive stimulation sessions;
- Integration of subjective self-report questionnaires with objective physiological measurements to enable a multidimensional assessment of relaxation;
- Development of a multimodal feature extraction and machine learning pipeline for the automatic classification of relaxation-related states from biosignals.

The remainder of the paper is organized as follows: Section 2 reviews related work on sound-based and vibroacoustic therapies and multimodal physiological monitoring; Section 3 describes the SonikB3D system and experimental methodology; Section 4 presents the experimental validation setup and results. Finally, Section 5 concludes the paper, and Section 6 discusses limitations and future research directions.

## 2. Related Works

The relationship between auditory stimulation, vibroacoustic therapy, and physiological regulation has been explored across several domains, including music therapy, affective computing, and multimodal human–computer interaction [15]. However, these research areas have largely evolved independently, and only a limited number of studies attempt to integrate immersive audio, vibroacoustic stimulation, and machine learning-based physiological analysis within a unified experimental framework.

### 2.1. Audio-Based Relaxation and Music Therapy

Auditory stimulation has long been used as a non-pharmacological intervention for stress reduction and emotional regulation. Music therapy studies consistently report reductions in perceived stress and changes in autonomic nervous system activity, often reflected in heart-rate variability, electrodermal activity, and EEG band power [13,14].

More recently, spatial and immersive audio technologies have been explored as a way to increase engagement and enhance the sense of presence during therapeutic listening [11]. Binaural and 3D audio rendering can improve spatial perception and immersion, which are hypothesised to modulate emotional responses more effectively than traditional stereo playback. Nevertheless, most studies rely primarily on subjective questionnaires or simple physiological indicators, and rarely attempt automatic or subject-independent classification of the induced physiological states.

### 2.2. Vibroacoustic Stimulation and Multisensory Approaches

Vibroacoustic therapy introduces low-frequency mechanical vibrations delivered through a bed or chair, typically synchronised with music. Prior research suggests that vibroacoustic stimulation may promote relaxation, reduce anxiety, and support rehabilitation in clinical and wellness contexts [8].

Despite promising results, the physiological mechanisms underlying these effects remain insufficiently characterised. Existing studies typically focus on pre–post comparisons or threshold-based indicators, rather than continuous monitoring or predictive modelling of user state. Furthermore, the interaction between vibroacoustic stimulation and immersive spatial audio has received limited experimental attention. Consequently, the combined multisensory effects of sound and vibration remain insufficiently understood.

### 2.3. Physiological Monitoring for Affective State Recognition

Affective computing has made significant progress in recognising emotional and stress-related states using physiological signals such as EEG, electrodermal activity (EDA), and photoplethysmography (PPG). Machine learning approaches have demonstrated the feasibility of classifying arousal, valence, stress, and relaxation from multimodal biosignals [18].

However, most datasets and experimental paradigms in this field rely on visual stimuli, cognitive tasks, or standardised emotional datasets. Comparatively few studies investigate physiological responses to immersive auditory or vibroacoustic environments. As a result, the generalisability of existing affect-recognition models to multisensory therapeutic environments remains largely unexplored.

### 2.4. Positioning of the Present Study

Previous work by our group introduced the SonikB3D platform [17] and demonstrated the feasibility of combining 3D audio, vibroacoustic stimulation, and physiological monitoring in music-relaxation sessions using threshold-based analysis. That preliminary study established experimental protocols and confirmed that measurable physiological changes occur during sessions [17]. The present work extends this line of research by moving from threshold-based assessment to machine learning analysis of multimodal physiological signals recorded during multisensory sessions. Rather than attempting to distinguish specific stimulation modes, this study focuses on evaluating whether consistent physiological patterns can be automatically detected across participants using subject-independent modelling. This represents a necessary step toward future adaptive and personalised multisensory therapeutic systems.

In this study, we therefore restrict quantitative analysis to a single stimulation protocol (binaural 3D audio combined with vibroacoustic stimulation), using it as a controlled test case for multimodal modelling, while leaving systematic comparisons between stimulation modes to future studies.

This step represents a necessary foundation for the development of future adaptive, closed-loop, and personalised multisensory therapeutic systems.

## 3. Methods

The development of the SonikB3D system followed three main phases: platform construction, sensor integration, and experimental evaluation. In the first phase, a modular wooden platform was designed and built, consisting of five independent compartments, three of which contained vibroacoustic transducers for transmitting low-frequency vibrations. Controllers, amplifiers, and signal processing modules were integrated to enable real-time generation and spatialization of both audible and tactile sound stimuli. The modular design allows flexible operation in different setups, including binaural and immersive 3D sound environments (surround sound). The second phase focused on integrating physiological monitoring systems to capture central and peripheral responses. EEG equipment was complemented by EDA and PPG sensors, ensuring a comprehensive physiological assessment. A dedicated software framework was developed for real-time synchronization and visualization of physiological data with sound stimulation, facilitating time-aligned analysis of multimodal signals. In the third phase, experimental sessions were conducted with volunteers exposed to controlled vibroacoustic and auditory stimuli. Participants completed validated questionnaires before and after each session, and physiological indicators (EEG, EDA, and PPG) were analyzed alongside subjective data. Preliminary results indicate a measurable shift toward relaxation, reflected by increased parasympathetic dominance, reduced electrodermal activity, and enhanced alpha and theta EEG activity.

### 3.1. Mechanical Structure and Electronics Development

**Figure 1:**
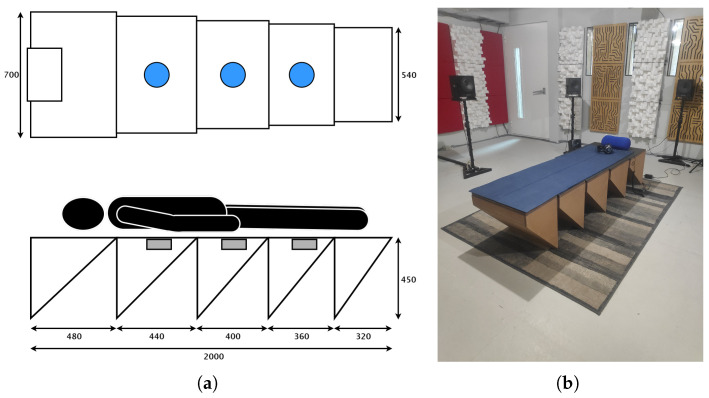
(**a**) Design diagram; (**b**) photo of the SonikB3D sound therapy system.

The physical infrastructure of the SonikB3D project consists of a platform that allows a person to lie down comfortably to participate in scheduled sessions to study the effects of sound and vibration on the human body and mind (see Figure 1).

**Figure 2:**
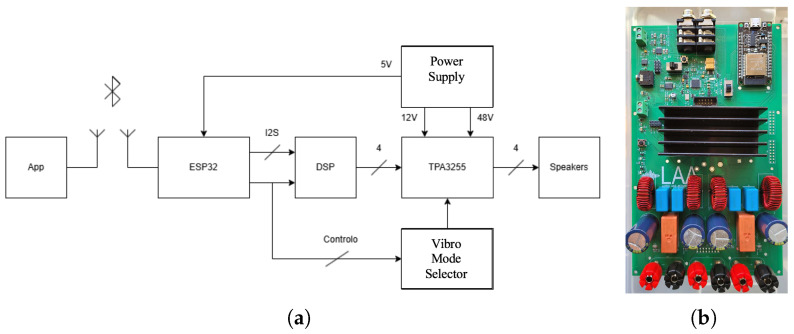
Block (**a**) Diagram of the excitation system of vibration devices; (**b**) electronic board of the excitation system of vibration devices.

This platform was developed in five separate compartments (connected by a snap-together system) to facilitate mobility, transportation, and installation in different locations. Each part consists of closed wooden boxes, designed to ensure structural robustness and function as acoustic enclosures with specific vibroacoustic characteristics. Vibroacoustic transducers are attached to some of the compartments. A Bluetooth music playback system utilizing a microcontroller (ESP32), a digital audio processor (DSP), and an audio amplifier to create an isolated and controlled sound environment was developed. The solution aims to combine efficiency, sound quality, and flexibility for use in different scenarios. The project block diagram is illustrated in Figure 2.

The main blocks of the project consisted of (i) a mobile application to control the ESP32 microcontroller via Bluetooth. This application is responsible for streaming audio from a predefined playlist (e.g., Spotify, VLC) and for controlling and parameterizing the entire system (built in FlutterFlow—Dart, provided by Google); (ii) Microcontroller ESP32—responsible for controlling the DSP signal processing modules (e.g., filter cutoff frequency and gain) and the mode switch to change the amplifier output configuration (OFF, Stereo (2 channels), and Quadro (4 channels) for the vibroacoustic transducer system. It also implements a state machine that allows one to control the operating sequence modes of the four vibroacoustic transducers (simultaneous, sequential forward or reverse, alternating); (iii) Digital Signal Processor (DSP): processes the signal used in the headphones (binaural—binaural beats hearing and vibroacoustic transducer frequency response compensation; (iv) a Multichannel amplifier—TPA3255 (https://www.ti.com/product/TPA3255, accessed on 1 June 2026)—to feed vibroacoustic transducer; (v) a Power Supply Module used to supply power to all modules (TPA3255—12 V and 54 V and the ESP32—5 V).

### 3.2. Sound and Vibro-Acoustic Stimuli for Relaxation

Auditory stimuli play a fundamental role in modulating emotional and physiological states. To test hypotheses and obtain the data necessary for analysis, different types of sensory stimuli and regimes were applied, focusing on auditory and tactile stimuli. These stimuli are chosen based on their ability to influence both brain activity and heart rate patterns, promoting states of calm and relaxation.

For this study, three main types of auditory stimuli were used:

- Sound Environments with 3D Sound: the acoustic stimuli consist of synthesized or real-world 3D soundscapes, either recorded in natural environments or created using spatial audio rendering. These soundscapes are designed to provide a three-dimensional sense of space and to enhance sensory immersion through realistic sound spatialization. Another stimulus was music selected based on its rhythmic and harmonic characteristics, known to induce relaxation [19]. Compositions with slow tempos, soft harmonies, and no sudden changes in volume or rhythm were used, such as classical, ambient, or meditation-specific music. The music is played through a 3D sound system or through high-quality headphones, ensuring an immersive experience free from external interference. Additionally, and simultaneously, the sound material was reproduced through the SonikB3D vibroacoustic transducer system, specially built for this purpose.
- Binaural Beats: These consist of tonal signals with slightly different frequencies presented to each ear, creating an illusion of a beat that can influence brain waves. These stimuli are often associated with inducing meditative states and deep relaxation [4]. Specific frequencies were used that promote an increase in theta and alpha waves, associated with relaxation (4.0–7.5 Hz—states of deep relaxation) and calm (7.5–12.0 Hz—light relaxation and concentration), respectively.
- Sound Mixing: Overlaying sound environments with 3D sound + binaural beats, offering an immersive auditory experience combined with neuronal induction. This approach is widely used in therapy sound libraries.
- Tactile Stimuli: In addition to auditory stimuli, tactile stimuli are applied to assess the response of the cardiovascular and neurological systems to physical sensations that promote relaxation. The intensity and frequency of the vibrations are adjusted to ensure they are comfortable and effective in inducing states of calm. The vibroacoustic bed is programmed to operate in different modes, varying the intensity and pattern of vibrations, allowing us to evaluate how different tactile stimuli influence the synchronization between brain and heart activity. These stimuli were applied in conjunction with auditory stimuli, allowing for an integrated analysis of how the combination of different sensory modalities can enhance the relaxation effects.

Among the available auditory and vibroacoustic configurations, the present study focuses on the protocol Sound Mixing, which combines 3D sound environments + binaural beats with synchronized vibroacoustic stimulation, which was used as the primary condition for quantitative analysis.

### 3.3. Experimental Environment

**Figure 3:**
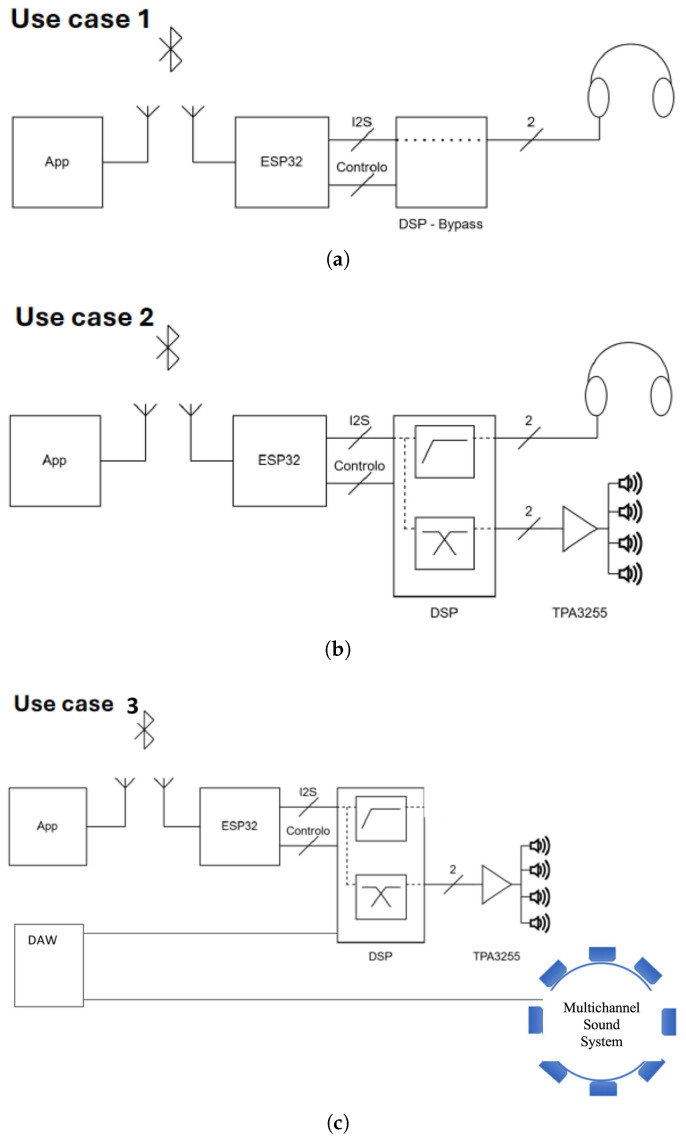
Use Cases used in sound therapy sessions with the SonikB3D platform: (**a**) Listening with headphones (Binaural Beats or Sound Environments with 3D Sound); (**b**) Listening with headphones + SonikB3D vibroacoustic bed; (**c**) Listening with vibration in the SonikB3D structure + multichannel sound system.

The stimuli were applied in controlled environments to ensure the accuracy and reliability of the collected data, specifically in the Immersive Sound Room of the Audio and Acoustics Laboratory (LAA) at ISEL (https://acusticaudiolab.isel.pt/). This room features high sound insulation and acoustic treatment suitable for sound listening. This ensures that there are no external interferences that could compromise the quality of the collected data and that participants have the best sound experience. The choice between the 3D sound system and headphones will depend on the need for immersion and the precision of stimulus delivery. The sessions were organized according to three Use Cases (as shown in Figure 3): (1) Listening with headphones (Binaural Beats or Sound Environments with 3D Sound); (2) Listening with headphones + SonikB3D vibroacoustic bed; (3) Listening with vibration in the SonikB3D structure + multichannel sound system (controlled by DAW—Digital Audio Workstation) in the Immersive Sound Room of the Audio and Acoustics Laboratory at ISEL.

Although the SonikB3D platform supports multiple stimulation use cases, in the present study all quantitative analyses, including machine learning experiments, were restricted to a single protocol combining binaural 3D audio stimulation with vibroacoustic stimulation delivered through the SonikB3D platform.

### 3.4. Acquisition Equipment

**Figure 4:**
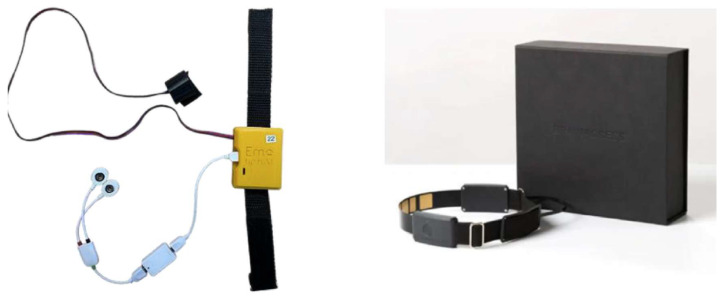
Physiological signal collection devices used in the SonikB3D project, (**left**) Scientisst-EMOTIPHAI sensor; (**right**) BrainAccess HALO.

Physiological data collection in the SonikB3D project is performed using non-invasive, portable devices compatible with the experimental environment illustrated in Figure 4.

The selection of equipment was guided by the need to obtain signals of sufficient quality for scientific analysis, while ensuring the comfort and safety of the participants. Peripheral signals (PPG and EDA) were acquired with the EMOTIPHAI sensor (https://www.scientisst.com/projects/emotiphai, accessed on 1 June 2026), a device designed to continuously collect physiological signals. The EMOTIPHAI measures the photoplethysmography signal, allowing the calculation of heart rate and heart rate variability (HRV), as well as the skin conductance signal, which reflects sympathetic activation. This device is easily attached to the participant’s arm and communicates via Bluetooth, making it ideal for sessions with limited freedom of movement, such as those conducted in a reclining position. Brain activity monitoring was performed with the BrainAccess HALO (https://www.brainaccess.ai/hardware/brainaccess-halo/, accessed on 1 June 2026), an EEG system that captures multichannel signals with reasonable temporal resolution (four channels—two frontal and two occipital). This device is lightweight, wireless, and easy to place, allowing reliable recordings under experimental conditions without the need for a complex laboratory environment. Its use allows the analysis of brain oscillations in specific frequency bands, such as alpha and theta, which are relevant in identifying states of relaxation.

To ensure reliable measurements and participant comfort, the EmotiBit was placed on the ring finger, using a soft spring, to monitor the PPG signal, and on the palm of the hand to record electrodermal activity (EDA). The BrainAccess HALO was carefully adjusted to the head, ensuring correct electrode placement and avoiding interference from movement or hair contact.

### 3.5. Physiological Background & Metrics

To objectively assess relaxation during the experimental sessions, we adopted a multimodal physiological monitoring framework. Three complementary biosignals were recorded and analysed: photoplethysmography (PPG), electrodermal activity (EDA), and electroencephalography (EEG). Together, these signals provide a combined view of autonomic and central nervous system activity associated with stress and relaxation.

When integrated, these measures offer a multidimensional view of autonomic and central nervous system dynamics, allowing objective validation of relaxation states during therapy sessions.

Photoplethysmography (PPG) employs optical sensors—typically placed on the fingertip or earlobe—to detect changes in blood volume in microvascular tissue. From the PPG waveform, inter-beat intervals (IBIs) are obtained, allowing the calculation of heart rate (HR) and heart rate variability (HRV) metrics [16,20]. HRV quantifies fluctuations in the temporal intervals between successive heartbeats and is a key indicator of autonomic balance [21]. Standard time-domain HRV measures include SDNN (standard deviation of NN intervals), which reflects overall HRV and is influenced by both sympathetic and parasympathetic activity, and RMSSD (root mean square of successive differences), which primarily reflects parasympathetic (vagal) tone and increases with relaxation and emotional calmness. In the frequency domain, the ratio of low-frequency (LF: 0.04–0.15 Hz) to high-frequency (HF: 0.15–0.4 Hz) power (LF/HF ratio) provides insight into sympathovagal balance. A lower LF/HF ratio suggests parasympathetic dominance, typical of relaxed states. Increases in HF power and RMSSD after an intervention are consistently linked to enhanced parasympathetic activation and relaxation.

Electrodermal activity (EDA) measures skin conductance, reflecting sympathetic nervous system activity via sweat gland responsiveness. EDA is commonly decomposed into a tonic skin conductance level (SCL) and phasic skin conductance responses (SCRs). Elevated SCL and frequent or high-amplitude SCRs are markers of sympathetic arousal, whereas decreases in these parameters after an intervention indicate reduced arousal and stress [10]. Real-time EDA monitoring allows assessment of emotional and physiological arousal dynamics, and post-session reductions in SCL and SCR frequency/amplitude are reliable indicators of relaxation.

Electroencephalography (EEG) provides insight into cortical activity associated with consciousness, attention, and emotional states by recording electrical oscillations across canonical frequency bands: delta, theta, alpha, beta, and gamma [10]. During relaxation, EEG typically shows increased alpha-band (8–13 Hz) power, especially over parietal and occipital regions, associated with restful wakefulness and inward focus, and concurrent reductions in beta activity (13–30 Hz), which is linked to active thinking and alertness. Advanced EEG metrics, such as alpha power, band-power ratios (e.g., alpha/beta), spectral coherence, or neurofeedback markers, provide detailed neural correlates of relaxation. Combining EEG with PPG and EDA enables a comprehensive analysis of synchronized central (cortical) and peripheral (autonomic) responses.

In this work, the multimodal validation protocol consisted of baseline recordings of PPG, EDA, and EEG prior to the stimulation session, followed by continuous monitoring during the intervention. Continuous data capture during stimulation allows tracking the evolution of physiological parameters over time. Post-session analysis focuses on identifying changes consistent with relaxation: increases in HRV indices (HF power, RMSSD, SDNN), a reduction in the LF/HF ratio, decreases in SCL and SCR activity, and heightened alpha activity in EEG. The convergence of these markers—parasympathetic dominance (higher HRV measures), sympathetic withdrawal (lower EDA activity), and cortical relaxation (increased alpha power)—is widely associated with relaxation responses.

The heuristic rules used later for window-level labels in the machine-learning pipeline were grounded in these well-established physiological patterns of sympathetic arousal and parasympathetic dominance.

This multimodal approach not only provides objective support for subjective reports but also facilitates personalised assessment and supports the development of adaptive relaxation protocols.

Table 1 summarizes the variation in the main parameters for the Stress and Relaxation states shown by the evidence in various experimental studies conducted with people [22].

### 3.6. Participants

Data from 20 participants (N = 20; 18–45 years; M = 23.4, SD = 8.99; 12 female) were included in the final analysis. The sample comprised 13 undergraduate students, 4 graduate students, and 3 university staff members. All participants were native Portuguese speakers, reported normal hearing, and had no history of neurological or psychiatric disorders. Participants were instructed to refrain from consuming coffee or tea for at least 3 h prior to the experiment.

All participants provided written informed consent in accordance with institutional ethics guidelines. The study was approved by the University Ethics Committee of Instituto Superior de Engenharia de Lisboa, Lisbon, Portugal (approval no. 04/CE/2026) and conducted in accordance with the Declaration of Helsinki and its subsequent amendments.

Participants were recruited via informal invitation from the Audio and Acoustics group at ISEL. Participation was voluntary and uncompensated.

Given the exploratory nature of this pilot study, a convenience sample of 20 participants was deemed sufficient to evaluate feasibility and to obtain preliminary effect-size estimates for future power calculations.

## 4. Experimental Validation

Relaxation induced by multisensory sound stimulation was evaluated through continuous multimodal physiological monitoring and self-reported questionnaires. Three complementary biosignals were recorded: photoplethysmography (PPG), electrodermal activity (EDA), and electroencephalography (EEG). Together, these signals provide a multidimensional representation of autonomic and central nervous system activity, enabling an objective assessment of relaxation responses.

### 4.1. Experimental Design

The study followed a within-subject repeated-measures design in which each participant was exposed to all stimulation conditions on separate experimental days. This approach reduces inter-individual variability and allows each participant to act as their own control. Three experimental use cases were investigated: (i) binaural audio stimulation, (ii) binaural audio combined with vibroacoustic stimulation, and (iii) immersive 3D audio combined with vibroacoustic stimulation. Each participant completed three independent sessions, with one use case per session. Sessions were scheduled on different days to minimize fatigue and carry-over effects. The order of conditions was kept consistent across participants as this study represents a pilot validation. Additionally, the fixed order of experimental conditions may introduce potential order effects, which future studies will address using randomized or counterbalanced designs.

Although the full protocol included three use cases, in the present manuscript we restrict quantitative analyses, including the machine learning experiments and the main statistical analyses, to the binaural 3D audio (Sound Mixing) plus vibroacoustic stimulation condition. This protocol was selected as a controlled test case for multimodal modelling, while systematic comparisons between use cases are left for future work.

### 4.2. Experimental Procedure

All sessions were conducted in a controlled laboratory environment with dim lighting and minimal external noise in the Immersive Sound Room of LAA. Each session followed a standardized procedure. Participants first completed a questionnaire evaluating their current emotional and relaxation state. A 3-min silent resting period was then recorded to establish a physiological baseline and stabilize cardiovascular and electrodermal activity. Participants were subsequently instructed to lie down comfortably on the SonikB3D bed, minimize voluntary movements, and relax; closing the eyes was recommended to enhance immersion.

EEG, EDA, and PPG signals were continuously recorded during the stimulation phase, at sampling frequencies of 250 Hz, 100 Hz and 1000 Hz, respectively, which lasted approximately 10 min. At the end of the session, participants completed the same questionnaire, enabling direct comparison of subjective states before and after stimulation. After each session, physiological signals were automatically processed to extract relevant features, which were later compared with questionnaire outcomes.

### 4.3. Signal Processing and Feature Extraction

Physiological recordings were segmented into overlapping windows of 120 s with 50% overlap to preserve temporal dynamics while ensuring robust feature estimation. From the PPG signal, inter-beat intervals were derived using peak detection, and standard heart rate variability metrics were computed, including mean heart rate, SDNN, RMSSD, low-frequency (LF) power, high-frequency (HF) power, and the LF/HF ratio. These features quantify autonomic balance and parasympathetic activation.

Artifact removal was applied, including rejection of windows with extreme motion/noise, based on signal quality assessment using statistical methods, and removal of blinking/artifacts in the EEG.

Electrodermal activity analysis captured both tonic and phasic components. The tonic skin conductance level represented slow-varying arousal, while the number of skin conductance responses quantified transient sympathetic activity. To reduce inter-subject variability, amplitude thresholds were normalized per session using robust scaling.

EEG signals were analyzed by computing spectral power in the delta, theta, alpha, beta, and gamma frequency bands. Relative band powers and the alpha/beta ratio were also extracted to capture cortical activation patterns associated with relaxation. All features were concatenated into a multimodal window-level feature vector and stored together with subject and session metadata.

### 4.4. Machine Learning Pipeline

A two-stage window-to-session classification framework was adopted to balance temporal resolution and robustness in a small-sample setting. In the first stage, window labels were generated using a deterministic rule-based scheme derived from HRV and EDA thresholds. Windows were labeled as Relaxed when LF/HF < 1, RMSSD exceeded the session median, and SCR count was ≤1, and as Aroused when LF/HF ≥ 1 or SCR count exceeded 3 [23]. These thresholds were chosen as pragmatic, trend-oriented criteria reflecting parasympathetic dominance and low sympathetic arousal, rather than as clinically validated cut-offs, and are therefore interpreted as heuristic rules for weak supervision rather than diagnostic boundaries.

Neutral windows were excluded from training to reduce label noise. A Random Forest classifier was trained using standardized multimodal features.

Model evaluation followed a Leave-One-Subject-Out protocol to ensure generalization to unseen participants and prevent subject-level data leakage. For each fold, the trained model generated relaxation probabilities for all windows of the held-out subject. In the second stage, window-level probabilities were aggregated into session descriptors, including the mean relaxation probability, the final window probability, and the temporal trend across the session. A logistic regression model was then used to classify sessions as Relaxed or Non-Relaxed based on aggregated features. Ground-truth session labels were derived from questionnaire score differences between post- and pre-session assessments. Performance was evaluated using accuracy, precision, recall, F1-score, and bootstrap-based 95% confidence intervals.

The main hyperparameters for the Random Forest were number of trees = 200 and $max_{depth}$ = None, and for the logistic regression model were L2 regularization with C=1.0 and the ‘lbfgs’ solver.

### 4.5. Results

Participants completed emotional and relaxation questionnaires before and after each session. Negatively worded items were inverted, normalized, and aggregated into a global score. Across participants, the global score increased by approximately 8% on average, indicating a consistent trend toward increased relaxation following stimulation. Statistical analysis showed a significant increase in self-reported relaxation (paired *t*-test: *t*(19) = 3.05, *p* = 0.010; Wilcoxon signed-rank test: *p* = 0.016), with a mean relative change of approximately 6.9% and a bootstrap 95% confidence interval of roughly [2.0%, 11.8%].

Physiological recordings showed consistent trends toward relaxation across representative sessions, including decreases in the LF/HF ratio, increases in SDNN and RMSSD, reductions in electrodermal activity, and increases in EEG alpha power and the Alpha/Beta ratio. These changes are consistent with parasympathetic activation and reduced sympathetic arousal.

The machine learning pipeline achieved a session-level accuracy of 80% and an F1-score of 0.857. The model showed high precision for the Relaxed class, indicating no false positives, although some relaxed sessions were missed. Given the limited sample size (N = 20), these findings should be interpreted as preliminary but encouraging, supporting the feasibility of multimodal physiological monitoring combined with machine learning for assessing relaxation induced by combined binaural audio and vibroacoustic stimulation.

### 4.6. Limitations

This study has several limitations that should be considered. First, the sample size is relatively small, which limits statistical power and generalizability, particularly for machine learning models trained on physiological data with high inter-subject variability. Second, the present pilot study focused on evaluating a combined multisensory experience and did not include unimodal control conditions (audio-only or vibroacoustic-only), nor a no-stimulation control condition with matched-duration passive rest. This limits the ability to disentangle the specific effects of auditory and vibroacoustic stimulation from non-specific factors such as time, expectancy, or habituation. Future work will incorporate these controlled baseline conditions to isolate the individual and combined contributions of auditory and tactile stimulation.

In addition, the machine learning labels relied on heuristic rules for window-level labels and on pre–post self-report differences for session-level labels. This constitutes a form of weak supervision and may introduce label noise, further limiting model generalizability.

## 5. Conclusions and Final Remarks

This work utilized SonikB3D, a multisensory platform designed to investigate relaxation through the combination of immersive 3D audio, vibroacoustic stimulation, and multimodal physiological sensing. Although the platform supports multiple stimulation scenarios, the present study focused on a single protocol combining binaural audio and vibroacoustic stimulation as a controlled test case.

A pilot study involving 20 participants was conducted using synchronized EEG, PPG, and EDA measurements together with self-reported relaxation scores. Results show a statistically significant increase in perceived relaxation after the stimulation (paired *t*-test = 3.05, *p* = 0.01), corresponding to an average 8% improvement in normalized relaxation scores.

In addition, a two-stage machine learning pipeline based on window-level Random Forest classification followed by session-level aggregation achieved an accuracy of 80% and an F1-score of 0.857 in distinguishing relaxation-related states from multimodal physiological signals. These findings suggest that the combined use of binaural 3D sound and vibroacoustic stimulation can produce measurable physiological and subjective changes associated with relaxation, and that such changes can be automatically inferred from multimodal biosignals.

However, the present study should be considered a pilot feasibility study. The relatively small sample size, the exploratory design, and the absence of unimodal and no-stimulation control conditions limit the generalizability of the findings. Larger studies are required to better characterize inter-individual variability and to validate and refine the proposed machine learning pipeline.

Future work will include experiments with larger and more diverse cohorts, the introduction of music-only, vibration-only, and passive-rest baseline conditions, and the development of more objective and standardized relaxation metrics. Ultimately, the SonikB3D platform may contribute to the development of personalized biofeedback and emotion-aware therapeutic technologies.

## 6. Future Developments

The present work represents an initial step toward understanding how combined immersive audio (including binaural and 3D configurations) and vibroacoustic stimulation influence physiological markers of relaxation. The most immediate future direction is the validation of the proposed framework through larger and more diverse participant cohorts, together with the inclusion of baseline and unimodal control conditions (audio-only and vibration-only). These experiments will enable a more robust assessment of inter-individual variability and improve the statistical reliability of the machine learning models ([18]).

Another important direction concerns the development of more objective and standardized relaxation metrics. Future studies will investigate the integration of additional physiological indicators and improved labeling strategies to reduce reliance on subjective self-reports and weak supervision.

From a computational perspective, future work will explore temporal and multimodal deep-learning approaches to better exploit physiological signals as time series. Recurrent architectures such as LSTM and GRU networks, as well as lightweight Transformer encoders, could capture transient responses, sustained trends, and long-range dependencies that are not fully represented by static summary features. Multimodal fusion strategies may further improve robustness by combining complementary information from autonomic and cortical signals.

Building on a biofeedback paradigm, we plan to investigate closed-loop adaptive stimulation, where auditory and vibroacoustic parameters are dynamically adjusted in real time according to ongoing physiological responses [24]. Such approaches could enable personalized and time-sensitive relaxation interventions while maintaining interpretability for research and clinical validation.

In the longer term, and pending validation in larger studies, the proposed framework may support research in areas such as digital therapeutics, wearable biofeedback systems, and immersive virtual reality relaxation environments. These directions remain exploratory but highlight the broader potential of multisensory and physiology-driven sound-based technologies.

## Figures and Tables

**Table 1:** Main parameters for the Stress and Relaxation states.

Goal	Example Analysis	Interpretation
Stress detection	↑ HR + ↓ HRV (SDNN and RMSSD) + ↑ EDA (SCL/SCR) + ↑ beta wave + ↓ Alpha/Beta (EEG)	Sympathetic activation
Relaxation detection	↓ HR + ↑ HRV (SDNN and RMSSD) + ↓ EDA + ↑ alpha and theta waves + ↓ beta + ↑ alpha/beta (EEG)	Parasympathetic dominance

## Data Availability

The datasets used for this study are available from the corresponding author upon reasonable request.

## References

[1] Thayer J.F., Lane R.D. (2008). Claude Bernard and the heart-brain connection: Further elaboration of a model of neurovisceral integration. Neurosci. Biobehav. Rev..

[2] Xu Z., Hu S., Xie F., Wei X., Peng Q., Chen T. (2025). Effect of group music therapy combined with routine nursing on emotional stability in inpatients with manic episodes. Medicine.

[3] Lutz A., Brefczynski-Lewis J., Johnstone T., Davidson R. (2008). Regulation of the Neural Circuitry of Emotion by Compassion Meditation: Effects of Meditative Expertise. PLoS ONE.

[4] Xiong J., Jiang X., Cai B., Zhao L., Zhang Q., Luo J. (2025). Binaural beats for perioperative anxiety and pain: A systematic review and meta-analysis. Complement. Ther. Med..

[5] Vincent V., Skaczkowski G., Hughes-Barton D., Gunn K.M. (2025). Effectiveness of Sound-Based Interventions for Improving Functional Outcomes in Children: A Systematic Review of the Evidence. Occup. Ther. Int..

[6] Goldman J. (2002). Healing Sounds: The Power of Harmonics.

[7] Kantor J., Vilímek Z., Vítězník M., Smrčka P., Campbell E.A., Bucharová M., Grohmannová J., Špinarová G., Janíčková K., Du J. (2022). Effect of low frequency sound vibration on acute stress response in university students—Pilot randomized controlled trial. Front. Psychol..

[8] Fooks C., Niebuhr O. (2024). Effects of Vibroacoustic Stimulation on Psychological, Physiological, and Cognitive Stress. Sensors.

[9] Wigram A.L. (1996). The Effects of Vibroacoustic Therapy on Clinical and Non-Clinical Populations. Ph.D. Thesis.

[10] Vaz M., Summavielle T., Sebastião R., Ribeiro R.P. (2023). Multimodal Classification of Anxiety Based on Physiological Signals. Appl. Sci..

[11] Naal-Ruiz N., Lee H., Alonso-Valerdi L., Ibarra-Zarate D.I. (2024). Data on neurophysiological and psychological responses to audio immersive experience in stereo and 3D audio formats. BMC Res. Notes.

[12] Rodero E., Rodríguez De Dios I. (2023). The 3D Sound Power of Immersion Processing and Psychophysiological Effects of Binaural versus Stereo Audio Stories. Media Psychol..

[13] Pillalamarri R., Shanmugam U. (2025). A review on EEG-based multimodal learning for emotion recognition. Artif. Intell. Rev..

[14] Zhang H., Zhou Q.Q., Chen H., Hu X.Q., Li W.G., Bai Y., Han J.X., Wang Y., Liang Z.H., Chen D. (2023). The applied principles of EEG analysis methods in neuroscience and clinical neurology. Mil. Med. Res..

[15] El Sayed B.B., Basheer M.A., Shalaby M.S., El Habashy H.R., Elkholy S.H. (2025). The power of music: Impact on EEG signals. Psychol. Res..

[16] Mather M., Thayer J. (2018). How heart rate variability affects emotion regulation brain networks. Curr. Opin. Behav. Sci..

[17] Preto Paulo J., Lourenço A., Vaz da Silva V., Fernandes A., Pires J., Sebastião T., Toledo P., Mengucci M. SonikB3D—3D Sound And Vibration Approach For Monitoring Physiological Parameters In Music Therapy. Proceedings of the 3o Simpósio de Acústica e Vibrações-2025.

[18] Khandelwal M., Sharma A. (2025). Machine Learning and Deep Learning Techniques to Detect Mental Stress Using Various Physiological Signals: A Critical Insight. Wiley Interdiscip. Rev. Data Min. Knowl. Discov..

[19] Campbell D.G. (1997). The Mozart Effect: Tapping the Power of Music to Heal the Body, Strengthen the Mind, and Unlock the Creative Spirit.

[20] Rudics E., Buzás A., Pálfi A., Szabó Z., Nagy A., Hompoth E.A., Dombi J., Bilicki V., Szendi I., Der A. (2025). Quantifying Stress and Relaxation: A New Measure of Heart Rate Variability as a Reliable Biomarker. Biomedicines.

[21] Kim H.G., Cheon E.J., Bai D.S., Lee Y.H., Koo B.H. (2018). Stress and Heart Rate Variability: A Meta-Analysis and Review of the Literature. Psychiatry Investig..

[22] Oomen P., Geffen R., Gentile D., Atassi N., Farran B., Fellas N., Braun C., Cuevas-Villanueva V., Nadiradze L., Mgvdliashvili I. (2024). Resonant Phenomena of Sound Waves and their Expression in Physiology. Open Sci. Framew..

[23] Shaffer F., Ginsberg J.P. (2017). An Overview of Heart Rate Variability Metrics and Norms. Front. Public Health.

[24] Schopp L., Starke G., Ienca M. (2025). Clinician perspectives on explainability in AI-driven closed-loop neurotechnology. Sci. Rep..

